# GPU-based detection of protein cavities using Gaussian surfaces

**DOI:** 10.1186/s12859-017-1913-4

**Published:** 2017-11-16

**Authors:** Sérgio E. D. Dias, Ana Mafalda Martins, Quoc T. Nguyen, Abel J. P. Gomes

**Affiliations:** 1 Universidade da Beira Interior, Av. Marques D’Ávila e Bolama, Covilhã, 6200-001 Portugal; 2 Instituto de Telecomunicações, Av. Marques D’Ávila e Bolama, Covilhã, 6200-001 Portugal

**Keywords:** GaussianFinder, Cavity detection, Pocket detection, Gaussian kernel function

## Abstract

**Background:**

Protein cavities play a key role in biomolecular recognition and function, particularly in protein-ligand interactions, as usual in drug discovery and design. Grid-based cavity detection methods aim at finding cavities as aggregates of grid nodes outside the molecule, under the condition that such cavities are bracketed by nodes on the molecule surface along a set of directions (not necessarily aligned with coordinate axes). Therefore, these methods are sensitive to scanning directions, a problem that we call cavity ground-and-walls ambiguity, i.e., they depend on the position and orientation of the protein in the discretized domain. Also, it is hard to distinguish grid nodes belonging to protein cavities amongst all those outside the protein, a problem that we call cavity ceiling ambiguity.

**Results:**

We solve those two ambiguity problems using two implicit isosurfaces of the protein, the protein surface itself (called inner isosurface) that excludes all its interior nodes from any cavity, and the outer isosurface that excludes most of its exterior nodes from any cavity. Summing up, the cavities are formed from nodes located between these two isosurfaces. It is worth noting that these two surfaces do not need to be evaluated (i.e., sampled), triangulated, and rendered on the screen to find the cavities in between; their defining analytic functions are enough to determine which grid nodes are in the empty space between them.

**Conclusion:**

This article introduces a novel geometric algorithm to detect cavities on the protein surface that takes advantage of the real analytic functions describing two Gaussian surfaces of a given protein.

## Background

Macromolecules (e.g., proteins, nucleic acids, etc.) are the building blocks of living beings. In particular, proteins are relevant for the cell chemistry inasmuch they perform a variety of different functions, such as catalysts, transporters, sensors, and regulators of cellular processes. Such functions depend on the interactions that establish with other entities in the cell, namely long entities like nucleic acids (e.g., DNA) and with small entities like nucleotides, peptides, catalytic substrates, and man-made chemicals. Thus, such interactions have some flavors, namely: protein-ligand, protein-protein, protein-DNA, and so forth. It is clear that these interactions involve both shape complementarity and physicochemical complementarity between a protein and any other fitting entity.

Nevertheless, this article does not focus on physicochemical complementarity. Instead, the focus is on detecting cavities on the protein surface where ligands (i.e., small molecules) may bind. The detection of protein cavities is instrumental as a first step to establish the shape complementarity between a protein and a ligand. As noted by Kawabata and Go [1], identifying cavities is one of the simplest ways to predict ligand binding sites on the protein surface. In this sense, protein cavities can be seen as *putative* binding sites of a given protein for ligands.

The algorithms to identify binding sites on a molecular surface are divided into four categories: geometry-based, energy-based, evolution-based, and hybrid approaches [2]. In this paper, we are focused on geometry-based algorithms. These geometric algorithms are divided into three sub-categories [1], namely grid-based, sphere-based, and tessellation-based algorithms. Nevertheless, recently a more fine-grained classification for these algorithms has been reported by Krone et al. [3] and Simões et al. [4], which also considers hybrid categories as, for example, grid-and-sphere and grid-and-surface methods. Furthermore, Simões et al. [4] consider three more primary categories, including the one concerning surface-based methods.

Taking into consideration that this paper describes a hybrid grid-and-surface method, let us briefly review those methods involving grids and surfaces. *Grid-based methods* are characterized by mapping a protein onto an axis-aligned 3D grid, using then a particular geometric criterion to detect cavities on the protein surface. Well-known geometric criteria are those based on *distance* [5, 6], *visibility* [7, 8], and *depth* [9, 10]. Most grid-based algorithms use a visibility criterion that indicates the blocked directions (and non-blocked directions) between opposed points on the protein surface. That is, the protein surface plays the role of the occluder for cavities. Unfortunately, visibility-based grid methods are not orientation-invariant. In other words, changing protein’s orientation may lead to an undetected cavity because its previously blocked scanning directions turn into unblocked ones. This cavity bounds’ ambiguity results from the difficulty of distinguishing grid nodes belonging to cavities from those that do not.

*Surface-based methods* build upon the analytic description of the molecular surface (e.g., solvent-excluded surface [11] and Gaussian surface [12, 13]) and its shape descriptors [14], namely solid angles [15] and curvatures [16], so that the surface is segmented into regions, some of which correspond to surface cavities. However, segmentations produced by shape descriptors have not proven to be effective in the detection of molecular cavities because the resulting segments may not match such cavities or tentative binding sites [12]. Zachmann et al. [17] and Natarajan et al. [16] tried to solve this problem by merging small segments into larger ones and determining larger segments using global shape descriptors, respectively. However, there is no evidence that such segments correspond to molecular cavities because no benchmarking analysis based on a ground-truth database of binding sites to evaluate the precision of those algorithms was carried out.

In turn, *grid-and-surface based methods* use a grid (or a lattice) together with at least a surface. Parulek et al. [18] proposed a method that combines a non-uniform lattice of randomly-generated points —which can be understood a generalization of grid-based techniques— and an implicitly-defined analytic surface defined by kernel functions to approximate the solvent-excluded surface (SES) [19]. The randomly-generated points inside the surface and those points outside such isosurface that are beyond a given distance relative to isosurface are discarded straight away; the remaining points are then subject to a mutual visibility test to retain those that are deemed to be cavity samples. Similarly, Krone et al. [8] use a Gaussian surface that better adjusts to SES, in conformity with the parameters set in [20] and [19]. But, instead of using sample points of the domain outside the surface, they used the vertices of the surface mesh triangles to test mutual visibility through an ambient occlusion-based visibility criterion due to Borland [21]. In both methods, the idea was to extract and track protein cavities in the context of molecular rendering and visualization, not on evaluating the accuracy of any cavity detection method relative to a certified ground truth.

As mentioned above, this paper addresses a grid-and-surface method, here called GaussianFinder. This method combines two Gaussian surfaces of a given protein, called inner and outer surfaces, as a way of finding cavities as clusters of voxels located between those two surfaces. As shown further ahead, this solves the ambiguity problems of grid-based methods mentioned above, i.e., the problems faced in the delineation of the limits of protein cavities, without using any visibility criterion of the grid-and-surface methods above to find cavities on the molecular surface. GaussianFinder aims at finding protein cavities accurately relative to ground-truth binding sites certified by known databases, as the one known as PDBsum (www.ebi.ac.uk/pdbsum/) [22].

Before proceeding any further, let us also mention the methods 3V and KVFinder due to Voss and Gerstein [23] and Oliveira et al. [10], respectively, resemble our method in solving the cavity ceiling and ground-and-walls ambiguities. But, while we find cavity voxels between two analytical surfaces, neither 3V nor KVFinder uses analytic surfaces to find such cavity voxels. Instead, they use probe and solvent spheres in conjunction with a grid, so they are grid-and-sphere methods [4]. 3V produces two voxelized volumes, the first of which is a discrete approximation to the solvent-excluded surface (SES), while the second approximates an inflated SES. The first voxelized volume is obtained after two steps. The first step collects all voxels inside atom-centered spheres whose radii are given by the van der Waals radii plus the water sphere radius of 1.5 Å, resulting in a voxelized volume that approximates the solvent-accessible surface (SAS). The second step discards voxels inside each solvent sphere centered at each frontier voxel of the SAS voxelized volume, resulting in a voxelized volume that approximates SES. This two-step procedure is repeated for the second voxelized volume, with the difference that one replaces the water sphere radius by a default probe sphere radius of 6.0 Å, so that the resulting voxelized volume approximates an inflated SES. Therefore, the cavity voxels are those that result from the difference between the second voxelized volume and the first voxelized volume that approximates SES.

In regards to KVFinder, one obtains the cavity voxels by the difference of two but different voxelized volumes. KVFinder uses a solvent sphere of radius 1.4 Å and a default probe sphere radius of 4.0 Å. However, this method only operates on grid points outside of the molecule atoms. In the first step, KVfinder collects all outside grid points such that the solvent sphere centered at each outside grid point fits in the empty outside space without overlapping the molecule. The second step is identical to the first one, with the difference that one uses the default probe sphere instead of the solvent sphere. The cavity voxels are those that belong to the first voxelized volume, but not to the second one. Therefore, cavity voxels correspond to the empty outside space where the solvent sphere gets in, but the default probe sphere does not.

## Implementation

### The Gaussian surface

GaussianFinder builds upon the concept of Gaussian surface, which is defined as the level set

1 $$  F(\mathbf{x}) = c  $$

where $F(\mathbf {x})={\sum \nolimits }_{i = 1}^{n}f_{i}$ is the summation of a number *n* of Gaussian kernel functions *f*_*i*_, one function per atom *i*, and $c\in \mathbb {R}$ is the isovalue. Each kernel function $f_{i}(\mathbf {x}):\mathbb {R}^{3} \rightarrow \mathbb {R}$ is given by the following expression:

2 $$  f_{i}(\mathbf{x}) = e^{-\beta\left(\frac{||\mathbf{x}-\mathbf{x}_{i}||^{2}}{r_{i}^{2}}-1\right)}  $$

where **x**_*i*_ and *r*_*i*_ stand for the center location and van der Waals radius of the *i*-th atom, respectively, while *β* represents the Gaussian kernel decay value. Therefore, the Gaussian surface depends on two parameters, *c* and *β* [24, 25].

### The leading idea

**Fig. 1:**
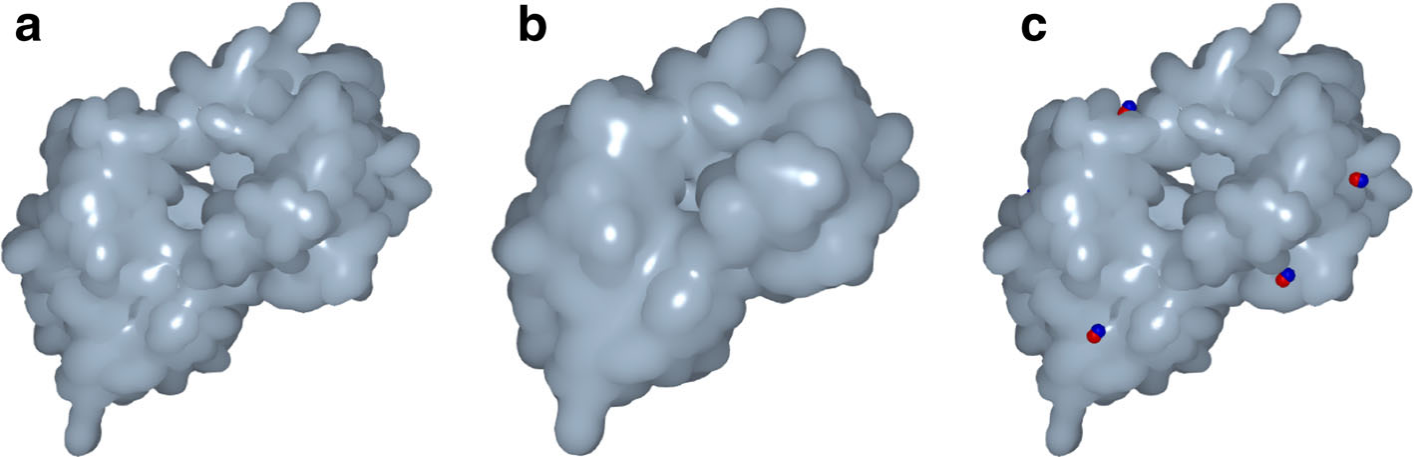
The protein 1A7X with 2155 atoms: (**a**) the inner surface; (**b**) the outer surface; (**c**) the inner surface with 4 out of 10 cavity locations determined by GaussianFinder (in red) and their homologous cavity locations set by the PDBsum ground truth (in blue)

GaussianFinder identifies cavity grid nodes between two Gaussian surfaces, *F*_*in*_(**x**)=*c* and *F*_*out*_(**x**)=*c* of each protein (see Fig. 1), which are defined by the following two functions:

3 $$  F_{in}(\mathbf{x})=\sum\limits_{i = 1}^{n} e^{-\beta\left(\frac{||\mathbf{x}-\mathbf{x}_{i}||^{2}}{r_{i}^{2}}-1\right)}  $$

and

4 $$  F_{out}(\mathbf{x})=\sum\limits_{i = 1}^{n} e^{-\beta\left(\frac{||\mathbf{x}-\mathbf{x}_{i}||^{2}}{R_{i}^{2}}-1\right)}  $$

where *R*_*i*_=*r*_*i*_+*w*_*i*_, with *w*_*i*_=1.4 Å standing for the radius of the water molecule. The idea is to find cavities between the inner and outer surfaces where one or more water molecules fit. Assuming the axis-aligned bounding box *D* enclosing the protein has been previously decomposed into equally-sized cubic voxels of length *Δ*=1.0 Å, the minimum size of a cavity is a boxed region of 3×3×3 voxels, i.e., a minimum volume of 3.0 Å^3^. Furthermore, the parameterization (*c*,*β*) was set to (1.0,2.3) for both inner and outer surfaces because it is the one that more closely approximates the solvent-excuded surface (SES) [20, 24, 26–28].

### The GaussianFinder method: overview

**Fig. 2:**
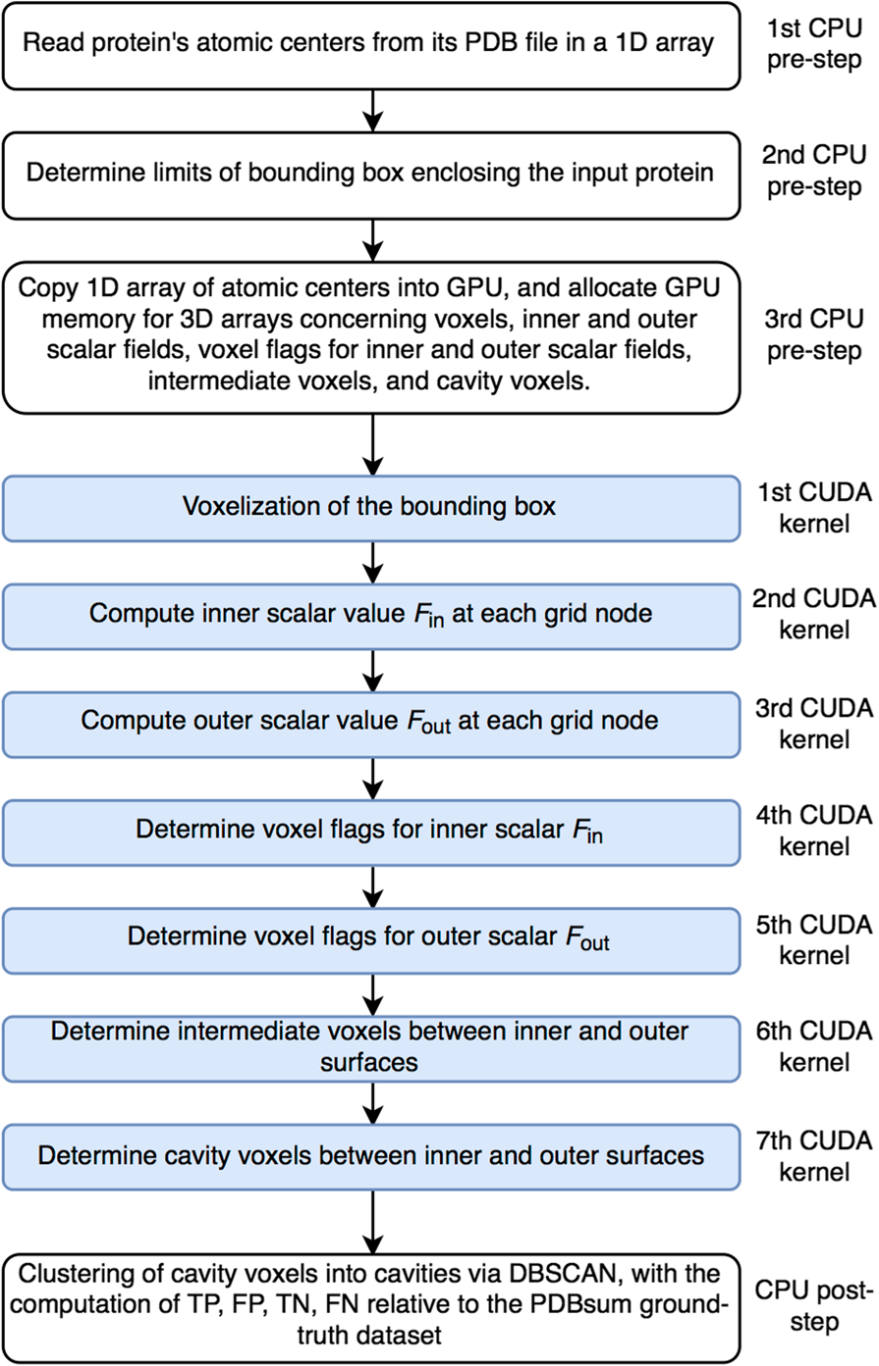
Flowchart of the GaussianFinder method
- Determine the bounding box $D \in \mathbb {R}^{3}$ that encloses the input protein on CPU side. This involves the computation of both minimum and maximum of the coordinates *x*, *y*, and *z* of the centers of all protein atoms, that is, the triples **p**=(*x*_*min*_,*y*_*min*_,*z*_*min*_) and **q**=(*x*_*max*_,*y*_*max*_,*z*_*max*_). These coordinates are then updated such that **p**=**p**−2*R* and **q**=**q**+2*R*, where *R* is the maximum atomic radius among the atoms belonging to the molecule, as needed to guarantee that the molecule lies in the box *D*.
- Copy the array of atomic centers into GPU memory, and allocate GPU memory for the following 3D arrays, as needed for: voxels of bounding box, *F*_*in*_, *F*_*out*_, intermediate voxels between the inner and outer surfaces, and cavity voxels. These 3D arrays of voxels are size-congruent and depend on the voxel length *Δ*=1.0.

The diagram of the GaussianFinder method is shown in Fig. 2. Before running the GaussianFinder on GPU, one performs three preprocessing steps as follows:

- Read atomic centers of a protein from the PDB file (http://www.rcsb.org) in an array on CPU side.

After completing the pre-processing stage, GaussianFinder identifies the cavities of an input protein through the following seven steps on GPU:

1. Voxelize the bounding box *D*, i.e., a grid of nodes.
2. Calculate *F*_*in*_(**x**) at every grid node.
3. Calculate *F*_*out*_(**x**) at every grid node.
4. Calculate voxel flags for *F*_*in*_(**x**).
5. Calculate voxel flags for *F*_*out*_(**x**).
6. Identify intermediate voxels (or grid nodes) between the inner and outer surfaces.
7. Identify cavity voxels among the intermediate voxels.

Note that the PDB file reading operation runs on CPU side. Then, the array of atomic centers (i.e., triples of coordinates *x*, *y*, and *z*) allocated in memory is transferred to GPU memory using the CUDA (Compute Unified Device Architecture [29]) function cudamemcpy. After that, the CUDA kernels encoding the GaussianFinder steps, a kernel per step, are ready to run on GPU one after another, as described below. However, the last step runs on CPU side using the DBSCAN algorithm [30], as needed to cluster cavity voxels into separate cavities.

### Voxelization of the bounding box – Kernel 1

This is the first CUDA kernel. The voxelization of the bounding box *D* consists in partitioning *D* into a grid of equally-sized voxels (i.e., cubes) of length *Δ*=1.0 Å. Considering that the voxels are all axis-aligned, it thus suffices using only the 0-th corner (also called node) of each voxel to represent it, because the remaining seven corners of a voxel are 0-th corners of its adjacent voxels. Therefore, it suffices to allocate a 3-dimensional array of such 0-th corners representing the voxels on GPU side; this array is named V. The location of each 0-th corner is also calculated on the GPU side.

### Computation of *F*_*in*_ – Kernel 2

This kernel launches *N* threads (i.e., the size of array V), one per 0-th corner. Each thread calculates the value of *F*_*in*_ (see Eq. (3)) at each corner in V. These function values are stored in a 3D array on GPU, called FIN, with the same size as V. But, before running this CUDA kernel on GPU, it is first necessary to allocate memory for FIN on GPU, as described in the third pre-processing step.

### Computation of *F*_*out*_ – Kernel 3

This kernel is identical to the previous one, with the difference that now we use another 3D array on GPU to hold the values of *F*_*out*_ (cf. Eq. (4)).

### Computation of voxel flags for *F*_*in*_ – Kernel 4

To determine the intermediate voxels between the inner and outer surfaces in Step 6, we need to find the voxels outside of the inner surface. For that purpose, we determine the 8-bit flag for each voxel of the scalar field *F*_*in*_. Each bit is associated with each voxel corner so that we have 2^8^=256 possible configurations for each voxel. If *F*_*in*_<*c* at a voxel corner, its bit takes the value 1; otherwise, it takes on the value 0. Therefore, the flag 11111111_2_=255_10_ indicates that the corresponding voxel is outside the inner surface because the value of *F*_*in*_ decreases with the distance to the protein. The flags are stored in a 3D array, called FLAGIN, which is of the same size as FIN.

### Computation of voxel flags for *F*_*out*_ – Kernel 5

This kernel is the same as the previous kernel, with the difference that now the computation of voxel flags is for *F*_*out*_ instead of *F*_*in*_. But, now we are interested in voxels whose flag is 00000000_2_=0_10_, that is, voxels inside of the outer surface. The flags are stored in a 3D array, called FLAGOUT, which is of the same size as FOUT.

### Identification of the intermediate voxels – Kernel 6

Based on the results of 4-th and 5-th kernels, an intermediate voxel (*i,j,k*) between the inner and outer surfaces is easily identified through the condition FLAGIN(*i,j,k*)=255 and FLAGOUT(*i,j,k*)=0, here called the intermediate condition.

### Identification of cavity voxels – Kernel 7

This kernel retrieves the set of cavity voxels from the set of intermediate voxels. Note that not all intermediate voxels are cavity voxels. The condition for an intermediate voxel being a cavity voxel is that it is surrounded by a 3×3×3 neighborhood of intermediate voxels. This is so because we have to guarantee a water molecule of radius 1.4 Å fits inside a cavity. Finally, the set of cavity voxels encoded into a 3D array called CAVITYVOXELMARK is copied back to CPU via the function cudaMemcpy3D to be processed by the DBSCAN clustering algorithm.

### Formation of protein cavities

The last step of the GaussianFinder runs on CPU. We use the DBSCAN clustering algorithm to separate cavity voxels into clusters featuring protein cavities. The code of DBSCAN is publicly available at https://github.com/gyaikhom/dbscan. The reader is referred to Ester et al. [30] for further details about DBSCAN.

### Molecular triangulation

The graphics visualization of each protein requires the triangulation of the Gaussian molecular surface defined by *F*_*in*_(**x**)=*c*. This triangulation is carried out entirely on GPU side using the variant of the marching cubes algorithm introduced by Dias and Gomes [31–34].

**Fig. 3:**
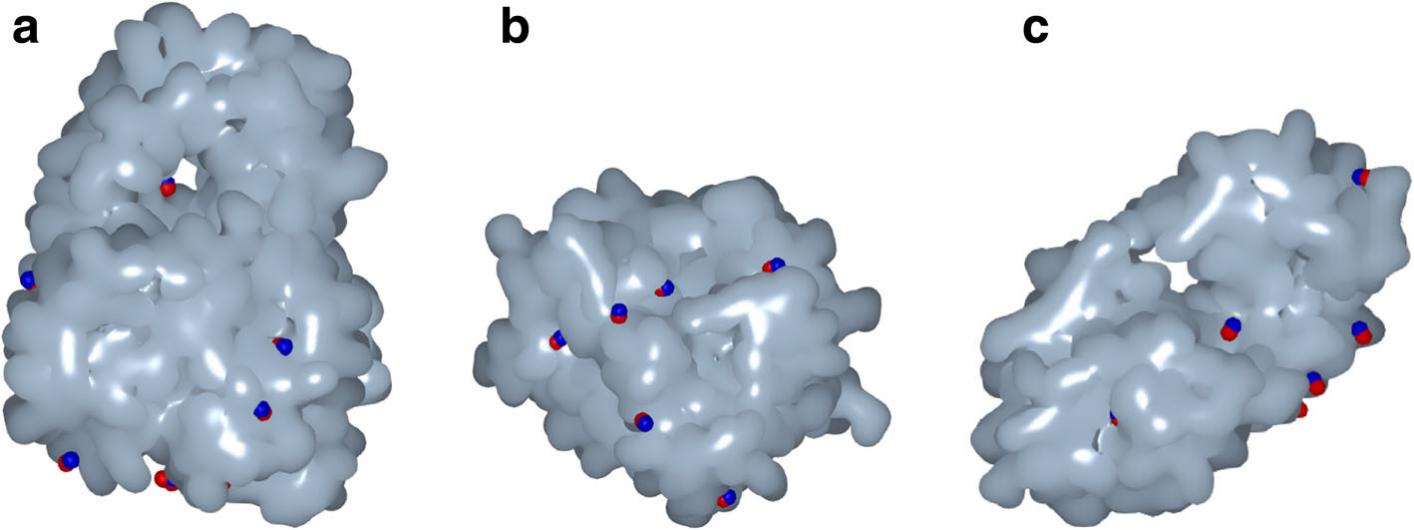
Gaussian surfaces and cavity locations determined by GaussianFinder (in red) and their homologous cavity locations set by the PDBsum ground truth (in blue) of: (**a**) the protein 1B2L with 1969 atoms and 2 out of 7 cavities; (**b**) the protein 1A58 with 1365 atoms and 3 out of 7 cavities; (**c**) the protein 148L with 1323 atoms and 4 out of 7 cavities

Figure 3 shows the Gaussian surfaces (in gray) of tree proteins after their triangulation, as well as some of their cavities, whose locations are identified by small balls in red, as determined by the GaussianFinder. The small balls in blue indicate the certified locations of the same cavities as given by the PDBsum ground truth. We see that there is a match between the locations of cavities calculated by our algorithm and those determined by PDBsum dataset.

## Results

The experimental testing results were obtained using a methodology built upon the following aspects: (i) hardware/software setup; (ii) a ground-truth dataset of protein cavities; (iii) set of benchmarking protein cavity detection methods; (iv) performance quality; (v) GPU time performance; and (vi) GPU memory space consumption.

### Hardware/software setup

Testing was accomplished using a desktop computer running the Linux Fedora 25 operating system and equipped with an Intel-Core I7 6800K 3.4GHz GHz Processor, 32GB RAM, one Nvidia Tesla K40, and one Nvidia Quadro M6000. Most computations to detect cavities of proteins and other molecules took place on the Nvidia Tesla K40. Also, all the computations needed to triangulate surfaces of molecules and their cavities were performed on the same Nvidia Tesla K40. The Nvidia Quadro M6000 was only used for graphics output and visualization.

Furthermore, GaussianFinder was written in C/C++ together with CUDA 9.0 to run on GPU. As noted above, we used the DBSCAN clustering algorithm to form clusters of cavity voxels featuring protein cavities. This clustering step runs on CPU side. Triangulating and rendering surfaces of proteins and binding sites on GPU were performed using a variant of the GPU-based implementation of the marching cubes algorithm by Dias and Gomes [31–34].

### Ground-truth dataset of protein cavities

We used PDBsum (www.ebi.ac.uk/pdbsum/) as the ground-truth dataset of protein cavities because it provides us with already known binding sites for a set of proteins [22]. In practice, we only used a subset of proteins in PDBsum; specifically, we used the dataset of proteins available in the LigASite database [35] which consists of 816 apo proteins and 1788 holo proteins, in a total of 2604 proteins. Recall that an apo protein is a protein without ligands, while a holo protein is a protein-ligand complex. The corresponding PDB files were retrieved from PDB Data Bank (www.rcsb.org). By inspection of the LigASite dataset in the PDBsum, we counted 8150 cavities on apo proteins, and 17850 cavities on holo proteins.

### Benchmarking cavity detection methods

For benchmarking sake with GaussianFinder, we used the following protein cavity detection methods:

- *POCASA*. It is essentially a grid-based method, called Roll, though it also uses a crust-like surface of probe spheres (see Yu et al. [36]).
- *SURFNET*. It includes the sphere-based method proposed by Laskowski [37].
- *PASS*. It includes the sphere-based method proposed by Brady et al. [38].
- *Fpocket*. It includes a triangulation-based method based on a Voronoi tessellation and alpha spheres on the top of a convex hull algorithm (see Guilloux et al. [39]).
- *GHECOM*. It includes the sphere-based method proposed by Kawabata [40].
- *ConCavity*. It includes the grid-based method proposed by Laskowski [41].
- *3V*. This grid-and-sphere method was proposed by Voss and Gerstein [23].
- *KVFinder*. This grid-and-sphere method was introduced by Oliveira et al. [10].

These methods and the GaussianFinder were run on the same desktop computer to guarantee a fair comparison between them. Note that the first six methods listed above are also part of Metapocket [42].

### Quality of performance

Let us now to analyze the performance quality of each benchmark cavity detection algorithms relative to the PDBsum ground-truth dataset of apo and holo proteins. For that purpose, we first counted 8150 cavities on the 816 apo proteins, and 17850 cavities on the 1788 holo proteins of the ground-truth dataset.

Then, upon execution of the DBSCAN, we extracted the number of clusters identified as cavities, here called positive cavities *C*_*P*_. These positive cavities include the true positive (TP) and false positive (FP) cavities (see Tables 1 and 2). We use the PDBsum ground-truth dataset, where the certified cavities are described per protein, to decide if a positive cavity outputted by DBSCAN is either a true positive or a false positive. Such a decision builds upon the *overlapping condition* which states that the geometric center of a protein cavity, as determined by a given benchmarking method, must be within a distance *d*∈[0.0,4.0]Å from the geometric center of the homologous cavity provided by the PDBsum ground-truth. For example, Table 1 shows the GaussianFinder was able to identify 8730 apo protein cavities within a maximum distance *d*=4.0Å, 7697 of which were correctly identified; that is, for GaussianFinder, *C*_*P*_=8730, *TP*=7697, and *FP*=*C*_*P*_−*TP*=1033.

**Table 1:** Performance of benchmarking detection methods for *apo* proteins in terms of: (*d*) distance (FN) false negatives to PDBsum ground-truth cavity centers; (TP) true positives; (FP) false positives; (TN) true negatives; (*S*_*v*_) sensitivity; (*S*_*c*_) specificity; (*a*) accuracy; (*r*_*d*_) ratio of detected ground-truth cavities; and (*C*_*u*_) cumulative number of undetected ground-truth cavities

	GaussianFinder	ConCavity	POCASA	SURFNET	PASS	GHECOM	Fpocket	3V	KVFinder
*d*∈[0.0,1.0]	7100	1512	2310	1737	4915	4562	5869	3751	4578
*d*∈]1.0,2.0]	188	107	176	1227	884	255	195	417	476
*d*∈]2.0,3.0]	227	127	226	520	117	380	207	291	305
*d*∈]3.0,4.0]	182	203	297	432	157	413	257	148	193
*TP*	7697	1949	3009	3014	6073	5610	6528	4607	5552
*FP*	1033	2103	863	902	325	581	878	1017	979
*TN*	2045	860	1697	1247	3086	2540	2827	1782	1889
*FN*	393	207	289	216	44	500	148	428	467
*S*_*v*_	0.951	0.904	0.912	0.933	0.993	0.918	0.978	0.915	0.922
*S*_*c*_	0.664	0.290	0.663	0.580	0.905	0.814	0.763	0.636	0.659
*a*	0.872	0.549	0.803	0.792	0.961	0.883	0.901	0.815	0.837
*r*_*d*_	0.944	0.239	0.369	0.369	0.745	0.688	0.801	0.565	0.681
*C*_*u*_	453	6201	5141	5136	2077	2540	1622	3543	2598

**Table 2:** Performance of benchmarking detection methods for *holo* proteins in terms of: (*d*) distance (FN) false negatives to PDBsum ground-truth cavity centers; (TP) true positives; (FP) false positives; (TN) true negatives; (*S*_*v*_) sensitivity; (*S*_*c*_) specificity; (*a*) accuracy; (*r*_*d*_) ratio of detected ground-truth cavities; and (*C*_*u*_) cumulative number of undetected ground-truth cavities

*d*	GaussianFinder	ConCavity	POCASA	SURFNET	PASS	GHECOM	Fpocket	3V	KVFinder
*d*∈[0.0,1.0]	16081	3133	5234	3668	11738	10438	14063	9174	12493
*d*∈]1.0,2.0]	366	239	338	2574	2100	571	432	703	719
*d*∈]2.0,3.0]	410	296	419	1063	281	789	406	811	813
*d*∈]3.0,4.0]	334	488	609	925	360	932	504	349	418
*TP*	17191	4156	6600	8230	14479	12730	15405	12049	14443
*FP*	2460	2155	2083	1806	634	1278	2151	1564	1941
*TN*	3231	916	2673	1559	4080	4968	3423	2476	2725
*FN*	440	362	214	227	207	658	391	511	553
*S*_*v*_	0.975	0.919	0.969	0.973	0.986	0.951	0.975	0.959	0.963
*S*_*c*_	0.568	0.298	0.562	0.463	0.866	0.795	0.614	0.612	0.584
*a*	0.876	0.668	0.801	0.828	0.957	0.901	0.881	0.875	0.873
*r*_*d*_	0.963	0.233	0.369	0.461	0.811	0.713	0.863	0.675	0.809
*C*_*u*_	659	13694	11250	9620	3371	5120	2445	5801	3407

Note that the maximum distance *d*=4.0 between geometric centers of homologous cavities has to do with the minimum size of a cavity, which in turn is related to the size of the water molecule. Most algorithms consider that the water molecule has a radius of 1.4 Å to 1.8 Å, so its diameter is 3.6 Å maximum. For example, Paramo et al. [43] use a 50 Å^3^ for the cavity’s minimum size, which corresponds to a cube length of 3.684 Å. Thus, a distance of 4.0 Å between the center of cavity detected by a given method and the center of its homologous cavity in the PDBsum ensures that such cavities extensively overlap, unless they are very small cavities. In fact, as Pérot et al. [44] noted, a drug-binding cavity has an average volume of about 930 Å^3^ when one uses a geometric-based method [14], and about 610 Å^3^ in the case of using an energy-based approach to detect pockets [45].

Finally, it is worth noting that DBSCAN rejects some clusters as cavities, here called negative cavities *C*_*N*_. These negative cavities include the true negative (TN) and false negative (FN) cavities (see Tables 1 and 2). So, we repeat the matching process between negative cavities and ground-truth cavities to decide which of them are not cavities truly (TN), and, consequently, those that are cavities but that were incorrectly classified as not (FN). For example, Table 1 shows that DBSCAN rejected 2438 clusters as cavities of apo proteins, 393 of which are cavities indeed; that is, for GaussianFinder, *C*_*N*_=2438, *T* *N*=2045, and *F* *N*=*C*_*N*_−*T* *N*=393.

**Fig. 4:**
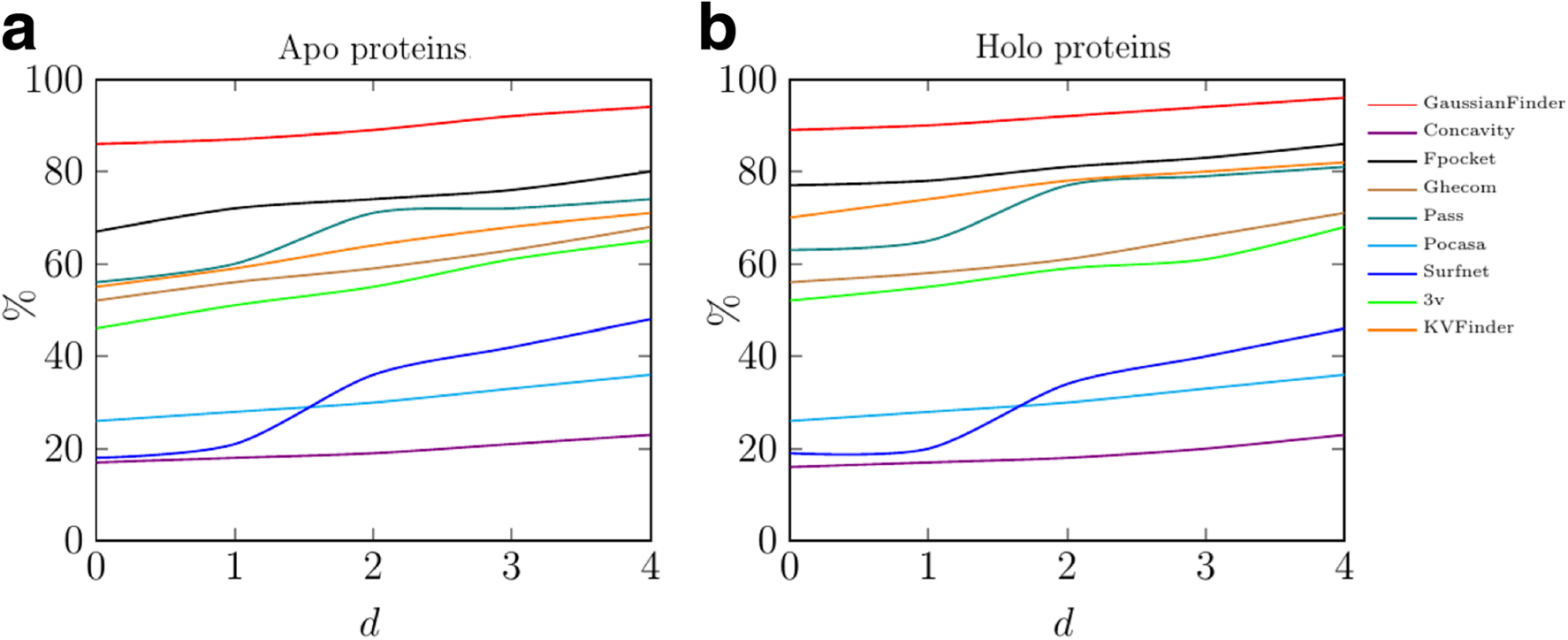
Cumulative cavity percentage (100. *r*_*d*_) of various detection methods in function of the distance *d* to ground-truth geometric centers for: (**a**) apo structures; and (**b**) holo structures

The performance quality of the predictions can be assessed using various metrics, namely: sensitivity or true positive rate $\left (S_{v}=\frac {TP}{TP+FN}\right)$, specificity or true negative rate $\left (S_{c}=\frac {TN}{TN+FP}\right)$, accuracy $\left (a=\frac {TP+TN}{TP+FP+FN+TN}\right)$, rate of detected ground-truth cavities $\left (r_{d}=\frac {TP}{C}\right)$, and undetected ground-truth cavities (*C*_*u*_=*C*−*T* *P*). Recall that the number of apo protein ground-truth cavities is *C*=8150, while *C*=17850 is the number of ground-truth cavities for holo proteins. From Tables 1 and 2, we observe that all methods have high values of sensitivity (*S*_*v*_>0.9), but GaussianFinder ranks behind PASS, GHECOM, and Fpocket regarding specificity because the value of *TN* is not much greater than the value of *FP*. However, these four methods possess an accuracy about 90% (*S*_*c*_≈0.9). Among these methods, GaussianFinder ranks first because its rate of detected ground-truth cavities (*r*_*d*_) stands out above the other methods (see Fig. 4). This means that GaussianFinder is more accurate than other benchmark methods relative to the number of detected ground-truth cavities. Note that the number *C*_*u*_ of undetected ground-truth cavities is far less for GaussianFinder than for any other method.

### Time performance

**Fig. 5:**
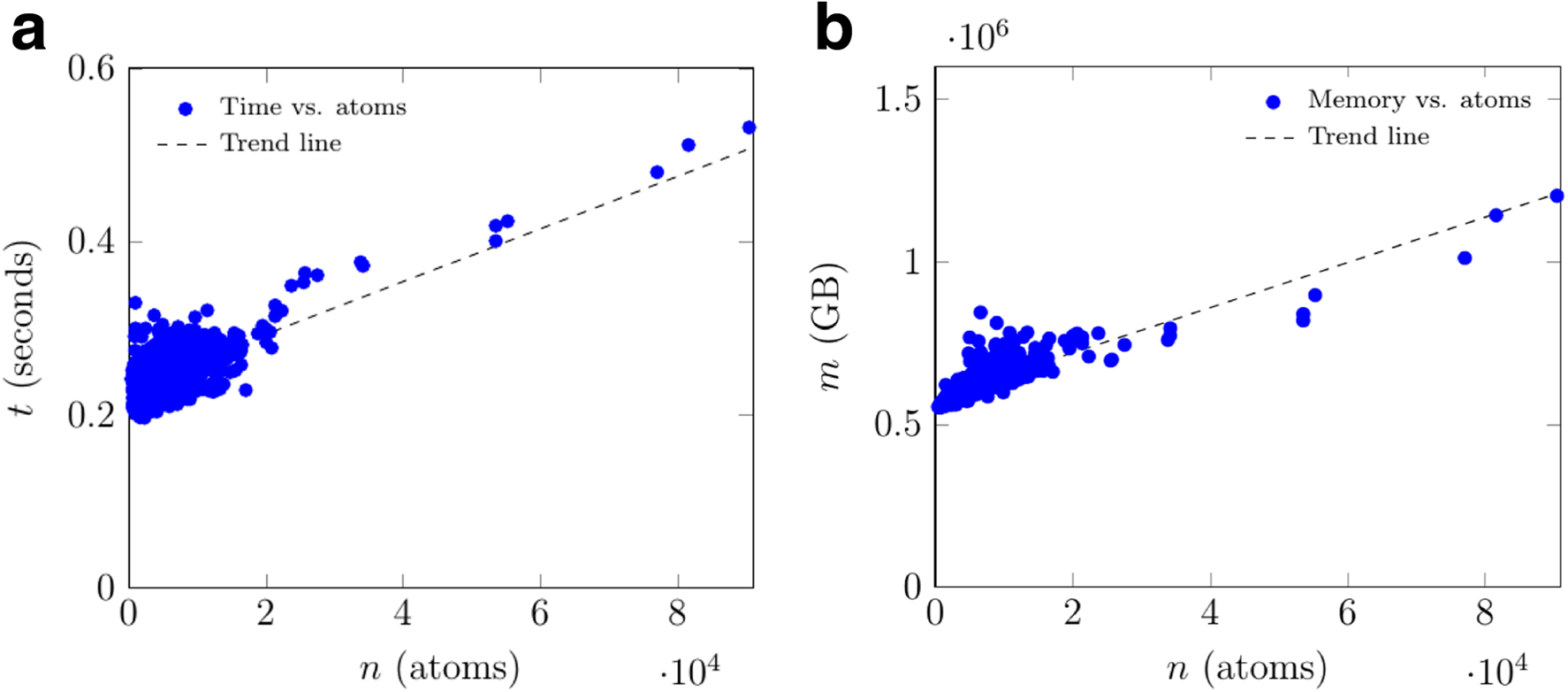
GaussianFinder on GPU: (**a**) experimental time performance; (**b**) experimental memory space occupancy

The experimental time performance of our cavity detection algorithm on GPU is shown in Fig. 5 a, whose (dashed) trend line satisfies the following expression:

5 $$ t=0.00000304n+0.232  $$

That is, the GaussianFinder runs in $\mathcal {O}(n)$ time. Eq. (5) was obtained by curve fitting [46]. Thus, the experimental time complexity of our method is linear on GPU. For example, finding the cavities of a molecule with 3000 atoms takes about 0.24 s GPU. For the entire set of proteins, the GaussianFinder takes 636.40 s (11 minutes approximately) to determine all the data needed to pass to DBSCAN algorithm to make the cavities of all proteins. These times are end-to-end GPU run-times, i.e., times needed to run the seven steps or kernels of the GaussianFinder.

### Memory space consumption

A brief glance at Fig. 5 b shows that the memory consumption is linearly related to the increase of the number of atoms. But the memory consumption of this algorithm is not very high when compared with other algorithms that also use a grid-based approach. This is so because the grid spacing (or voxel length) is 1.0 Å for GaussianFinder. It is clear that a smaller grid spacing would consume much more memory space on GPU.

## Discussion

In light of previous results, also depicted in Fig. 4, we summarize our findings as follows. In our experiments, GaussianFinder seemingly outperforms all other cavity detection methods. Additionally, grid-based methods (ConCavity, and POCASA) are less accurate than sphere-based methods (SURFNET, PASS, and GHECOM) in our test conditions; in turn, sphere-based methods are less accurate than triangulation-based methods (Fpocket). In regards to the grid-and-sphere methods, we observe that KVFinder ranks third together with GHECOM, just behind Fpocket, while 3V performs not so well, but even so with a cumulative cavity percentage above 60%. Note that we used default parameters to obtain those results; for example, 3V uses the default radii of 1.5 Å and 6.0 Å for solvent and probe spheres, respectively, while KVFinder’s default radii are 1.4 Å and 4.0 Å, respectively.

Furthermore, every single benchmark geometric method tends to detect most cavities in the first interval [0.0, 1.0]. Also, every single benchmark method performs better for holo proteins than for apo proteins. Note that, in our tests, we only considered geometric detection methods for cavities (i.e., tentative binding sites). Moreover, we used actual locations of binding sites of proteins (via PDBsum) as the ground-truth for the cavities detected by those benchmarking methods, including GaussianFinder.

## Conclusions

We have introduced a novel grid-and-surface based algorithm, called GaussianFinder, for identifying cavities on protein surfaces without using a visibility criterion. The leading idea of the method is to determine the grid nodes between two Gaussian isosurfaces of each molecule, which are then aggregated into clusters of nodes featuring cavities. This avoids possible geometric ambiguities (concerning the limits of cavities) inherent to the use of grid-based methods to detect cavities of the protein surface. GaussianFinder is considerably fast, with the cavity detection stage finishing in a matter of a few seconds on a GPU-based workstation equipped with a Nvidia Tesla K40 and a Nvidia Quadro M6000. Shortly, we intend to parallelize other cavity detection algorithms existing in the literature for a more comprehensive comparison between algorithms in terms of time performance.

## Availability and requirements

**Project name:** GaussianFinder;

**Project home page:** https://sourceforge.net/projects/gaussianfinder;

**Operating system(s):** Linux Fedora 25;

**Programming language:** C/C++;

**Other requirements:** CUDA 9.0;

**Any restrictions to use by non-academics:** The source code is freely available under the GPLv3 License.

## Abbreviations

CUDA: Compute unified device architecture
CPU: Central processing unit
DNA: Deoxyribonucleic acid
GPU: Graphics processing unit
SES: Solvent-excluded surface

## References

[1] Kawabata T, Go N (2007). Detection of pockets on protein surfaces using small and large probe spheres to find putative ligand binding sites. Protein Struct Funct Bioinforma.

[2] Volkamer A, Griewel A, Grombacher T, Rarey M (2010). Analyzing the Topology of Active Sites: On the Prediction of Pockets and Subpockets. J Chem Inf Model.

[3] Krone M, Kozlíková B, Lindow N, Baaden M, Baum D, Parulek J, Hege HC, Viola I (2016). Visual Analysis of Biomolecular Cavities: State of the Art. Comput Graph Forum.

[4] Simões T, Lopes D, Dias S, Fernandes F, Pereira J, Jorge J, Bajaj C, Gomes A. Geometric detection algorithms for cavities on protein surfaces in molecular graphics: a survey. Comput Graph Forum. 2017. doi:10.1111/cgf.13158. 10.1111/cgf.13158 PMC5839519 29520122

[5] Voorintholt R, Kosters MT, Vegter G, Vriend G, Hol WG (1989). A very fast program for visualizing protein surfaces, channels and cavities. J Mol Graph.

[6] Zhang X, Bajaj C (2007). Extraction, quantification and visualization of protein pockets. Proceedings of the Computational Systems and Bioinformatics Conference (CSB’2007).

[7] Levitt DG, Banaszak LJ (1992). POCKET: A computer graphics method for identifying and displaying protein cavities and their surrounding amino acids. J Mol Graph.

[8] Krone M, Reina G, Schulz C, Kulschewski T, Pleiss J, Ertl T (2013). Interactive extraction and tracking of biomolecular surface features. Comput Graph Forum.

[9] Kalidas Y, Chandra N (2008). PocketDepth: A new depth based algorithm for identification of ligand binding sites in proteins. J Struct Biol.

[10] Oliveira SH, Ferraz FA, Honorato RV, Xavier-Neto J, Sobreira TJ, de Oliveira PS (2014). KVFinder: steered identification of protein cavities as a PyMOL plugin. BMC Bioinformatics.

[11] Zhu H, Pisabarro MT (2011). MSPocket: an orientation-independent algorithm for the detection of ligand binding pockets. Bioinformatics.

[12] Dias SED, Nguyen QT, Jorge JA, Gomes AJP (2017). Multi-GPU-based detection of protein cavities using critical points. Futur Gener Comput Syst.

[13] Gomes A, Voiculescu I, Jorge J, Wyvill B, Galbraith C (2009). Implicit Curves and Surfaces: Mathematics, Data Structures, and Algorithms.

[14] Nayal M, Honig B (2006). On the nature of cavities on protein surfaces: Application to the identification of drug-binding sites. Proteins.

[15] Connolly M (1986). Measurement of protein surface shape by solid angles. J Mol Graph.

[16] Natarajan V, Wang Y, Bremer PT, Pascucci V, Hamann B (2006). Segmenting molecular surfaces. Comput Aided Geom Des.

[17] Zachmann CD, Heiden W, Schlenkrich M, Brickmann J (1992). Topological analysis of complex molecular surfaces. J Comput Chem.

[18] Parulek J, Turkay C, Reuter N, Viola I (2012). Implicit surfaces for interactive graph based cavity analysis of molecular simulations. Proceedings of the 2012 IEEE Symposium on Biological Data Visualization (BioVis’2012).

[19] Richards FM (1977). Areas, volumes, packing, and protein structure. Annu Rev Biophys Bioeng.

[20] Grant JA, Pickup BT (1995). A Gaussian description of molecular shape. J Phys Chem.

[21] Borland D (2011). Ambient occlusion opacity mapping for visualization of internal molecular structure. J WSCG.

[22] Laskowski RA, Hutchinson GE, Michie AD, Wallace AC, Jones ML, Thornton JM (1997). PDBsum: a web-based database of summaries and analyses of all PDB structures. Trends Biochem Sci.

[23] Voss NR, Gerstein M (2010). 3v: cavity, channel and cleft volume calculator and extractor. Nucleic Acids Res.

[24] Blinn JF (1982). A generalization of algebraic surface drawing. ACM Trans Graph.

[25] Chowdhury R, Rasheed M, Keidel D, Moussalem M, Olson A, Sanner M, Bajaj C (2013). Protein-protein docking with f2dock 2.0 and gb-rerank. PLoS ONE.

[26] Gabdoulline RR, Wade RC (1996). Analytically defined surfaces to analyze molecular interaction properties. J Mol Graph.

[27] Zhang Y, Xu G, Bajaj C (2006). Quality meshing of implicit solvation models of biomolecular structures. Comput Aided Geom Des.

[28] Bajaj CL, Chowdhury R, Siddahanavalli V (2011). *f*^2^dock: Fast fourier protein-protein docking. IEEE/ACM Trans Comput Biol Bioinforma.

[29] Cook S (2012). CUDA Programming: A Developer’s Guide to Parallel Computing with GPUs.

[30] Ester M, Kriegel HP, Sander J, Xu X (1996). A density-based algorithm for discovering clusters a density-based algorithm for discovering clusters in large spatial databases with noise. Proceedings of the Second International Conference on Knowledge Discovery and Data Mining (KDD’96), Portland, Oregon, USA, August 2-4.

[31] Dias S, Bora K, Gomes A (2010). CUDA-based triangulations of convolution molecular surfaces. Proceedings of the 19th ACM International Symposium on High Performance Distributed Computing.

[32] Dias S, Gomes A (2011). Graphics processing unit- based triangulations of Blinn molecular surfaces. Concurr Comput Pract Experience.

[33] Dias S, Gomes AJP. Computational Electrostatics for Biological Applications In: Rocchia W, Spagnuolo M, editors. Cham: Springer: 2015. p. 177–98.

[34] Dias SED, Gomes AJP (2015). Triangulating molecular surfaces over a LAN of GPU-enabled computers. Parallel Comput.

[35] Dessailly BH, Lensink MF, Wodak SJ (2008). LigASite: a database of biologically relevant binding sites in proteins with known apo-structures. Acid Nucleic Res.

[36] Yu J, Zhou Y, Tanaka I, Yao M (2010). Roll: a new algorithm for the detection of protein pockets and cavities with a rolling probe sphere. Bioinformatics.

[37] Laskowski RA (1995). SURFNET: A program for visualizing molecular surfaces, cavities, and intermolecular interactions. J Mol Graph.

[38] Brady J, Patrick G, Stouten PW (2000). Fast prediction and visualization of protein binding pockets with PASS. J Comput Aided Mol Des.

[39] Le Guilloux V, Schmidtke P, Tuffery P (2009). Fpocket: An open source platform for ligand pocket detection. BMC Bioinformatics.

[40] Kawabata T (2010). Detection of multiscale pockets on protein surfaces using mathematical morphology. Proteins.

[41] Capra JA, Laskowski RA, Thornton JM, Singh M, Funkhouser TA (2009). Predicting protein ligand binding sites by combining evolutionary sequence conservation and 3d structure. PLOS Comput Biol.

[42] Huang B (2009). MetaPocket: A meta approach to improve protein ligand binding site prediction. OMICS.

[43] Paramo T, East A, Garzón D, Ulmschneider MB, Bond PJ (2014). Efficient characterization of protein cavities within molecular simulation trajectories: trj_cavity. J Chem Theory Comput.

[44] Pérot S, Sperandio O, Miteva MA, Camproux AC, Villoutreix BO (2010). Druggable pockets and binding site centric chemical space: a paradigm shift in drug discovery. Drug Discov Today.

[45] An J, Totrov M, Abagyan R (2005). Pocketome via comprehensive identification and classification of ligand binding envelopes. Mol Cell Proteome.

[46] Arlinghaus S (1994). Practical Handbook of Curve Fitting.

